# An *in vitro* assay to quantify satellite cell activation using isolated mouse myofibers

**DOI:** 10.1016/j.xpro.2021.100482

**Published:** 2021-04-23

**Authors:** Rodrigo Canibano-Fraile, Emma Boertjes, Stela Bozhilova, W.W.M. Pim Pijnappel, Gerben J. Schaaf

**Affiliations:** 1 Department of Clinical Genetics, Erasmus MC University Medical Center, 3015 GD Rotterdam, the Netherlands; 2 Department of Pediatrics, Erasmus MC University Medical Center, 3015 GD Rotterdam, the Netherlands; 3 Center for Lysosomal and Metabolic Diseases, Erasmus MC University Medical Center, 3015 GE Rotterdam, the Netherlands

**Keywords:** Cell isolation, Cell-based Assays, Microscopy, Stem Cells

## Abstract

Isolated myofibers offer the possibility of *in vitro* study of satellite cells in their niche. We describe a mouse myofiber isolation assay to assess satellite cell activation by quantifying myofiber-derived satellite cell progeny. The assay allows isolation of myofibers from a mouse using standard equipment and reagents. It can be used to compare satellite cells across different mouse models or to evaluate their response to treatments, offering a valuable complementary tool for *in vitro* experimentation.

## Highlights

• An *in vitro* assay to study satellite cell activation
• An optimized protocol for myofiber isolation
• Protocol enables comparison of satellite cell dynamics across different disease models
• Versatile 96-well format allows studying of multiple experimental conditions in parallel

## Graphical Abstract

**Figure:**
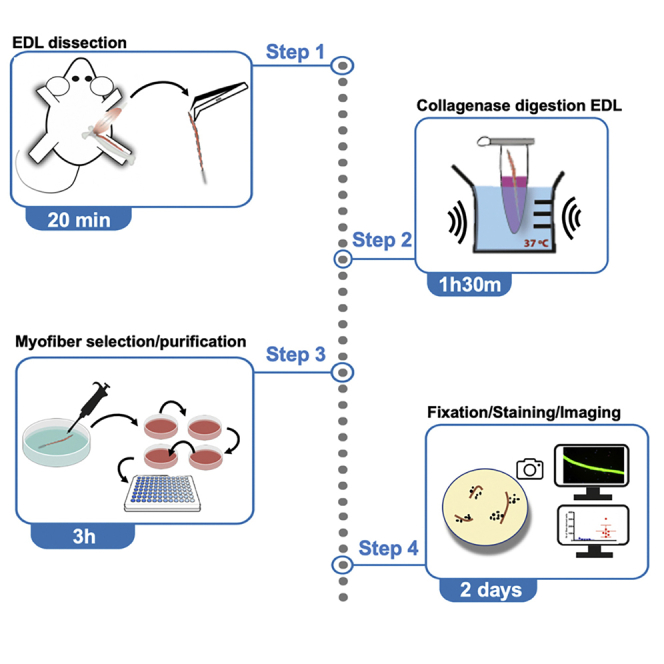

## Before you begin

***Note:*** All animal experiments were approved by the local Animal Experiments Committee and national Central Committee for Animal Experiments (animal experiment authorities in compliance with the European Community Council Directive guidelines (EU directive 86/609), regarding the protection of animals used for experimental purposes. All procedures with the animals were performed with the aim of ensuring that discomfort, distress, pain, and injury would be minimal.

### Prepare in advance

**Timing: 1 – 2 h (Optional: prepare one day before)**

***Note:*** Working under clean conditions is important to reduce the risk of contaminations. If possible work inside a laminar flow cabinet. As indicated below, EDL dissection and fiber selection/purification steps were performed outside the flow cabinet as the setup with the dissection microscope and plate warmer did not fit inside the flow cabinet. All other steps were performed inside a laminar flow cabinet. To allow working outside a laminar flow environment, clean all working surfaces and tools with 70% ethanol (EtOH) and desinfect pipet tips before use, sequentially in 70% EtOH, PBS, and horse serum (HS) (see Optional, below step-by-step method details step 10c). The media used in this protocol contain antibiotics (10,000 units penicillin and 10 mg streptomycin per mL) to minimize bacterial growth.

1. Prepare digestion solution. Weigh collagenase type II (Col II; 1000 U/mL in dilution medium (see below); aliquot per mL; in our hands it can be stored at −80°C for up to 6 months with negligible loss of yield; 2 × 1 mL Col II per mouse) (See troubleshooting – problem 1).

***Note:*** The use of collagenase is critical for correct tissue digestion. Other collagenase types have been reported to work (e.g., collagenase type I) (Brun, Wang and Rudnicki, 2018). This protocol has been optimized using collagenase type II (see key resources table).

***Note:*** Processing fibers from one mouse (i.e., 2 EDLs) takes 7–8 h per researcher, including plating fibers under experimental conditions.

2. Prepare media (use within 2 weeks after preparation) (See troubleshooting – problem 3). See materials and equipment for the procedures:
  a. Fiber selection medium. 100 mL per mouse. Store at 4°C.
  b. Proliferation medium. 10 mL per 96 well plate. Store at 4°C.
  c. Experiment base medium. 10 mL per 96 well plate. Store at 4°C.
  d. Dilution medium. 500 mL. Store at 4°C.
3. Coat 96-well plates with 5% ECM (v/v):
  a. Cover culture surface with cold ECM (typically 30 μL per well in 96 well format). Incubate at 4°C for 30 min. ECM can be re-used twice if kept cold through the whole process. Keep 5% solution on ice and store at 4°C.
  b. Remove ECM solution and incubate coated plate(s) at 37°C for 30 min or 12–16 h at 18°C–22°C.

***Optional:*** coat for 2^nd^ time: Repeat 3a-b.

***Note:*** Coating for a second time may improve the adherence of fibers. Its use is advised if fibers do not adhere well to the bottom of the plates.

### Before the experiment

**Timing: 1 h**

4. Incubate 4 × 100 mm cell culture dishes per EDL (8 dishes per mouse) with 100% horse serum (HS) for 30 min at 37°C. This will prevent fibers adhering to the dishes during the purification procedure.
5. Label dishes 1-1, 1-2, 1-3, 1-4 for EDL 1; and 2-1, 2-2, 2-3, 2-4 for EDL 2.
6. Replace HS with 10 mL of fiber selection medium for the first 3 dishes of each EDL (1-1, 1-2, 1-3, and 2-1, 2-2, 2-3).
7. Return the dishes to 37°C until use.
8. Replace HS with 10 mL of experiment base medium for dishes 1-4 and 2-4. This is done to prevent altering the experiment medium composition by mixing it with fiber selection medium (See step-by-step method details steps 11g–h).
9. Prewarm Col II solution to 37°C in Eppendorf tube heater.
10. Prewarm Slide Warmer.

***Note:*** To calibrate the temperature settings of the slide warmer place a 6-well-plate with medium and adjust temperature setting so that medium temperature remains 37°C. The slide warmer that was used in this protocol was set to 40°C. The slide warmer will be used to keep the dishes warm during fiber selection or during medium changes to avoid fiber contraction. Dishes with fibers should not be kept outside of the incubators for longer than 10 min despite using the slide warmer.

11. Prepare a disinfected working space, work if possible in a laminar flow cabinet.
  a. Spray surgical area with 70% EtOH.
  b. Disinfect surgical tools using 70% ethanol: 1 × fine-tip forceps (Extra Fine #5, DBIO), 1× blunt serrated forceps (Standard Forceps, DBIO), 1× scissors (Standard Pattern - Sharp/Blunt, DBIO), 1× fine scissors (Slim iris, DBIO), 1× surgical knife (Disposable Sterile Scalpel 11, Swann-Morton).
  c. 4× 25G needles (Sterican 100, Braun).
12. Prepare experiment plates; time spent on this step is largely dependent on the number of plates and treatments (approximately 30–60 min for 2 EDL muscles when plating all viable myofibers).
  a. Coat 96 well plates (Corning 96-well flat-bottom tissue culture plate) or polymer-based 96-well tissue culture plates allowing imaging (Nunc Nunclon 96-well plate with lid, Electron Microscopy Sciences) with 1:20 diluted ECM. We recommend at least 10 wells per treatment/genotype myofiber.
  b. Dilute experiment additives (i.e., growth factors, inhibitors, agonists, SiRNA) in experiment base medium to twice the final concentration (2× [Conc]_final_) in sterile Eppendorf tubes. The correct concentrations will be achieved after adding the fibers (See Note under step-by-step method details step 12c).
  c. Distribute 50 μl/well of the prepared experiment media over the appropriate wells. Fill outer wells with PBS to minimize evaporation of medium from treatment wells.
  d. Keep 96 well plate with pre-prepared experiment medium at 4°C until 15 min before plating (See step-by-step method details step 12a).
13. Collect equipment:
  a. Dissecting microscope (Olympus SZX16 was used for this purpose).
  b. 20–200 μl and 100–1000 μl pipettes.
  c. Sterile 200 and 1000 μl unfiltered tips and Eppendorf tubes.
  d. Scissors (to cut tips, clean and EtOH sterilize).
  e. Slidewarmer (Slidewarmer SW85 - Adamas Instruments or equivalent).
  f. Eppendorf tube heater (Eppendorf Thermomixer R for this purpose).

## Key resources table

**Table:** 

REAGENT or RESOURCE	SOURCE	IDENTIFIER
**Antibodies**		

Mouse Anti-chicken PAX7 (IgG1)	DSHB	RRID: AB_528428
Rabbit Anti-KI67	Abcam	Cat#: ab15580 RRID: AB_443209
Mouse Anti-MYOD (IgG2B)	Santa Cruz	Cat#: sc377460 RRID: AB_2813894
Biotinylated Anti-Mouse IgG (H+L), made in horse	Vector Labs	Cat#: BA2000 RRID: AB_2313581
Biotin Rat Anti-Mouse IgG1	BD Biosciences	Cat#: 553441 RRID: AB_394861
Goat anti-Rabbit AF488	Invitrogen	Cat#: A-11034 RRID: AB_2576217
Cy3-AffiniPure Goat Anti-Mouse IgG, Fcγ Subclass 2b Specific	Jackson ImmunoResearch	Cat#: 115-165-207 RRID: AB_2338696
Streptavidin, Alexa Fluor 647 conjugate	Thermo Fisher	Cat#: S21374

**Chemicals, peptides, and recombinant proteins**		

Dulbecco’s phosphate-buffered saline	Sigma-Aldrich	Cat#: D1408
DMEM	Lonza	Cat#: 12-614F
HAM's F10	Lonza	Cat#: BE12-618F
Fetal calf serum	Sigma-Aldrich	Cat#: FBS-12A
Horse serum	Gibco	Cat#: 16050-122
Bovine serum albumin (BSA)	Sigma-Aldrich	Cat#: 3294-100G
Tween-20	Sigma-Aldrich	Cat#: P1379
Triton X-100	Sigma-Aldrich	Cat#: X100
Penicillin streptomycin	Sigma-Aldrich	Cat#: P0781
Chicken embryo extract	US Biological	Cat#: C3999
KnockOut Serum Replacement	Invitrogen	Cat#: 10828010
Basic FGF	PeproTech	Cat#: 100-18B
Extracellular matrix	Sigma-Aldrich	Cat#: E1270
Collagenase Type II	Gibco	Cat#: 17101015
Paraformaldehyde	Sigma-Aldrich	Cat#: P-6148
Hoechst 33342	Invitrogen	Cat#: H3570

**Experimental models: cell lines**		

Isolated myofibers	N/A	N/A

**Experimental models: organisms/strains**		

Mouse: FVB/NHsd of both sexes, 40 weeks of age	Envigo	RRID:MGI:6112021
Mouse: GAAKO in FVB/N background of both sexes, 40 weeks of age	Bijvoet, A. G. A. *et al.* (1998)	N/A

**Software and algorithms**		

Zen 2011 (black edition) v7.0.0.285	Zeiss	N/A

**Other**		

Standard forceps	DBIO	Cat#: DBF1011
Extra Fine #5 Forceps	DBIO	Cat#: DBF1001
Slim Iris	DBIO	Cat#: DBS1001
Standard pattern - sharp/blunt	DBIO	Cat#: DBS1009
Disposable Sterile Scalpel 11	Swann-Morton	Cat#: 0511
Pipetman 20–200 μL	Gilson	Cat#: FA10005M
Pipetman 200–1000 μL	Gilson	Cat#: FA10006M
EasyLoad Universal 200 μL (pipette tip)	Greiner Bio-One	Cat#: 741065
EasyLoad Universal 1000 μL (pipette tip)	Greiner Bio-One	Cat#: 741035
15 mL Conical tubes	Thermo Fisher	Cat#: 339650
Olympus SZX16	Olympus	Cat#: SZX16
Nikon Eclipse Ti-E	Nikon	Cat#: Eclipse Ti-E
Slide warmer SW85	Adamas Instruments	Cat#: 39589585
Eppendorf Thermomixer R	Eppendorf	Cat#: 05-400-205
Sterican 25G Needles	Braun	Cat#: 465-7853
Corning 96-well flat-bottom tissue culture plate	Corning	Cat#: CLS3595
Nunc Nunclon 96-well plate with lid	Electron Microscopy Sciences	Cat#: 64810-05

## Materials and equipment

**Table:** 

Reagent	Used in text	Final concentration	Solvent	Storage
Phosphate Buffer Saline	PBS	N/A	N/A	4°C; >1 year
DMEM	DMEM	N/A	N/A	4°C; 1 month
Ham’s F10	Ham’s F10	N/A	N/A	4°C; 1 month
Fetal Calf Serum	FCS	User-defined	User-defined	−20°C; 1-12 months
Horse Serum	HS	User-defined	User-defined	−20°C; 1-12 months
Penicillin-Streptomycin	Pen-Strep	100 U/mL	PBS	−20°C; 1-12 months
Chicken Embryo Extract	CEE	1% (v/v)	User-defined	4°C; 1–6 months
Knockout Serum Replacement	KSR	5% (v/v)	Experiment base medium	−20°C; 1-12 months
Basic FGF	FGF2	20 ng/mL	PBA	−80°C; 1-12 months
Extracellular Matrix	ECM	5%	Dilution medium	4°C; 1 month
Collagenase Type II	Col II	1000 U/mL	Dilution medium	−20°C; 1-12 months
Paraformaldehyde	PFA	8% (w/v), NaOH (to dissolve); pH 7.0	PBS	−20°C; 1-12 months
PBS-BSA	PBA	0.1% BSA (w/v)	PBS	4°C; 1 month
PBS-BSA-Tween 20	PBA-Tw	0.1% BSA (w/v) 0.1% Tw20 (v/v)	PBS	4°C; 1 month
PBS-Tween 20	PBS-Tw	0.1% Tw20 (v/v)	PBS	4°C; 1 month
PBS-Triton X-100	Triton	0.5 Triton X-100 (v/v)	PBS	4°C; 1 month
Hoechst	Hoechst	1 μg/mL	PBS	4°C; >1 year

Fiber selection medium

**Table:** Store at 4°C for maximum 1 month.

Solution	Volume	Final concentration
DMEM	445 mL	-
FCS	25 mL	5%
HS	25 mL	5%
Penicillin-Streptomycin	5 mL	1%
**Total**	**500 mL**	**-**

Proliferation medium

**Table:** Store at 4°C for maximum 1 month.

Solution	Volume	Final concentration
Ham’s F10	390 mL	-
FCS	100 mL	20%
CEE	5 mL	1%
Penicillin-Streptomycin	5 mL	1%
**Total**	**500 mL**	**-**

Experiment base medium

**Table:** Store at 4°C for maximum 1 month.

Solution	Volume	Final concentration
DMEM	440 mL	-
HS	25 mL	5%
KSR	25 mL	5%
CEE	5 mL	1%
Penicillin-Streptomycin	5 mL	1%
**Total**	**500 mL**	**-**

Dilution medium

**Table:** Store at 4°C for maximum 1 month.

Solution	Volume	Final concentration
DMEM	470 mL	-
FCS	25 mL	5%
Penicillin-Streptomycin	5 mL	1%
**Total**	**500 mL**	**-**

**CRITICAL:** Paraformaldehyde (PFA) is toxic after swallowing or inhalation, causes skin irritation, harmful to eyes and respiratory tract; a potential carcinogen. Use in a safety cabinet or with sufficient ventilation. Wear protective measures (gloves, protective eye wear, facemask).

## Step-by-step method details

### Dissecting extensor digitalis longus

**Timing: 20 min per mouse**

In this step of the protocol EDL muscles from a mouse will be dissected as source of myofibers. This protocol is optimized for muscles that have clear identifiable tendinous insertions at both ends. Recently, a method that allows isolation of fibers from sources without tendinous ends has been published (Feige, Tsai and Rudnicki, 2021). We have successfully isolated fibers from soleus muscles using this protocol. Others have reported isolating myofibers from flexor digitorum brevis (FDB; (Garcia-Pelagio, Pratt and Lovering, 2020), but we have not tried this. The surgery is a critical step; therefore, the strategy needs to be optimized for muscles other than EDL. Stretching the target muscles will significantly decrease the yield of viable fibers. In addition, speed is an important factor, since allowing the muscle(s) to cool excessively will also decrease yield. For those starting with this protocol, practice EDL dissections on surplus mice (i.e., mice euthanized in other experiments) prior to the experiment are advised.

***Note:*** These steps can be performed outside the laminar flow cabinet.

**Figure 1:**
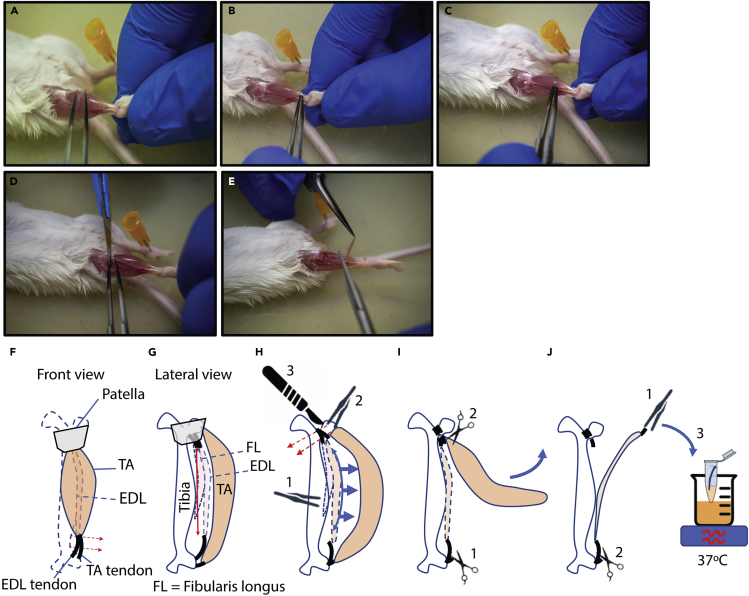
Dissecting the EDL (A) Removing fascia; (B) Inserting fine-tip forceps between distal tendons; (C) Liberate EDL/TA from tibial bone; (D) Cutting proximal EDL tendon; (E) Removing EDL; (F-J) Schematic representation of the dissection steps. (F) Inserting fine-tip forceps between distal tendons; (G) Liberating EDL/TA from tibial bone; (H) Liberating TA from EDL (1), inserting forceps behind proximal tendons (2), and sectioning cutting proximal EDL tendon with a scalpel (3); (I) Cutting distal part of TA muscle (1), cutting proximal part of TA muscle (2) and removing TA; (J) Transferring EDL to digestion solution. Lift EDL at proximal tendon (1) and cut distal tendon to release EDL (2). The number in the pictures indicate the order of events.

1. Euthanize mouse in line with institutional regulations. Here animals were killed by cervical dislocation.
2. Disinfect the hindlimbs with 70% EtOH.
3. Open the skin of one of the hindlegs to expose the lower leg muscles.
4. Remove the fascia covering the TA muscle from knee to ankle using the fine-tip forceps (Figure 1A).

**CRITICAL:** Remove as much fascia as possible, as remaining fascia will complicate the next step. If fascia are not sufficiently removed it will require more force to liberate the TA and EDL from the underlying bone and from each other. Applying too much force may damage or stretch the muscle and may decrease the yield and viability of isolated fibers .

5. Free the TA/EDL muscles from the bone.
  a. Insert the fine-tip forceps behind the ankle-tendons of both the EDL and TA (Figures 1B and 1F).
  b. Liberate the EDL and TA muscles from the underlying tibial bone by moving the forceps behind the EDL/TA up and down from knee to ankle (Figures 1C and 1G).
6. Liberate the TA from the EDL.
  a. Insert the fine tip forceps between the EDL and TA ankle tendons and lift up the TA and carefully move the forceps up towards the knee (Figure 1H).
  b. Expose the proximal EDL and fibularis longus tendons by removing tissue around the patella.
  c. Insert the fine tip forceps behind the proximal EDL and *fibularis longus* tendons.
  d. Cut the proximal EDL tendon using a scalpel blade (Figures 1D and 1H).
7. Remove TA muscle.
  a. Cut the TA distal tendon (near the ankle; Figure 1I).
  b. Lift the TA at the distal tendon.
  c. Cut TA proximal to the knee to remove it (Figure 1I). The EDL muscle is now exposed.

8. Remove the EDL muscle.
  a. Carefully lift the EDL at the proximal tendon.
    **CRITICAL:** Prevent stretching the EDL during handling (See troubleshooting – problem 1).
  b. Remove the EDL by cutting the distal (ankle) tendon (Figures 1E and 1J). Make sure to cut as distal as possible to ensure cutting the tendon, not the muscle .
  c. Place the EDL into a 1.5 mL tube with 1 mL of prewarmed Col II solution and shake (500 rpm) at 37°C for 1.5 h. in an Eppendorf Thermomixer (See troubleshooting – problem 1).

9. Repeat step-by-step method details steps 3–7 for the remaining EDL.
  ***Note:*** For some steps it may be easier to turn the animal 180° (head facing towards you) for dissecting the contralateral EDL.
  **CRITICAL:** To increase the yield of intact myofibers ensure to (See troubleshooting – problem 2):
  a.Work fast to prevent excessive cooling of EDL.
  b.Avoid damaging the EDL when liberating the muscles at step-by-step method details steps 5 and 6.
  c.Avoid stretching the EDL when removing it from the animal.

### Purifying intact myofibers

**Timing: 3 h**

***Note:*** For adult FVB/N mice we usually get 150–250 fibers per EDL. Nevertheless, the yield of fibers per EDL will vary depending on the mouse model. For example, disease models with muscle damage may yield lower number of fibers compared with healthy mice (see expected outcomes).

In these steps the myofibers are liberated from the digested EDL muscle and subsequently purified by sequential transferring intact myofibers into clean dishes.

***Note:*** These steps were performed outside a laminar flow cabinet. Prepare 3 × 15 mL conical tubes to disinfect and wash the utensils. 1 tube with 70% EtOH, 1 tube with PBS, and 1 tube with HS. For each use the pipet tips were cleansed by sequentially pipetting up/down 3 times in EtOH, PBS and HS. HS is to prevent fibers sticking to the walls of the pipet tips.

**Figure 2:**
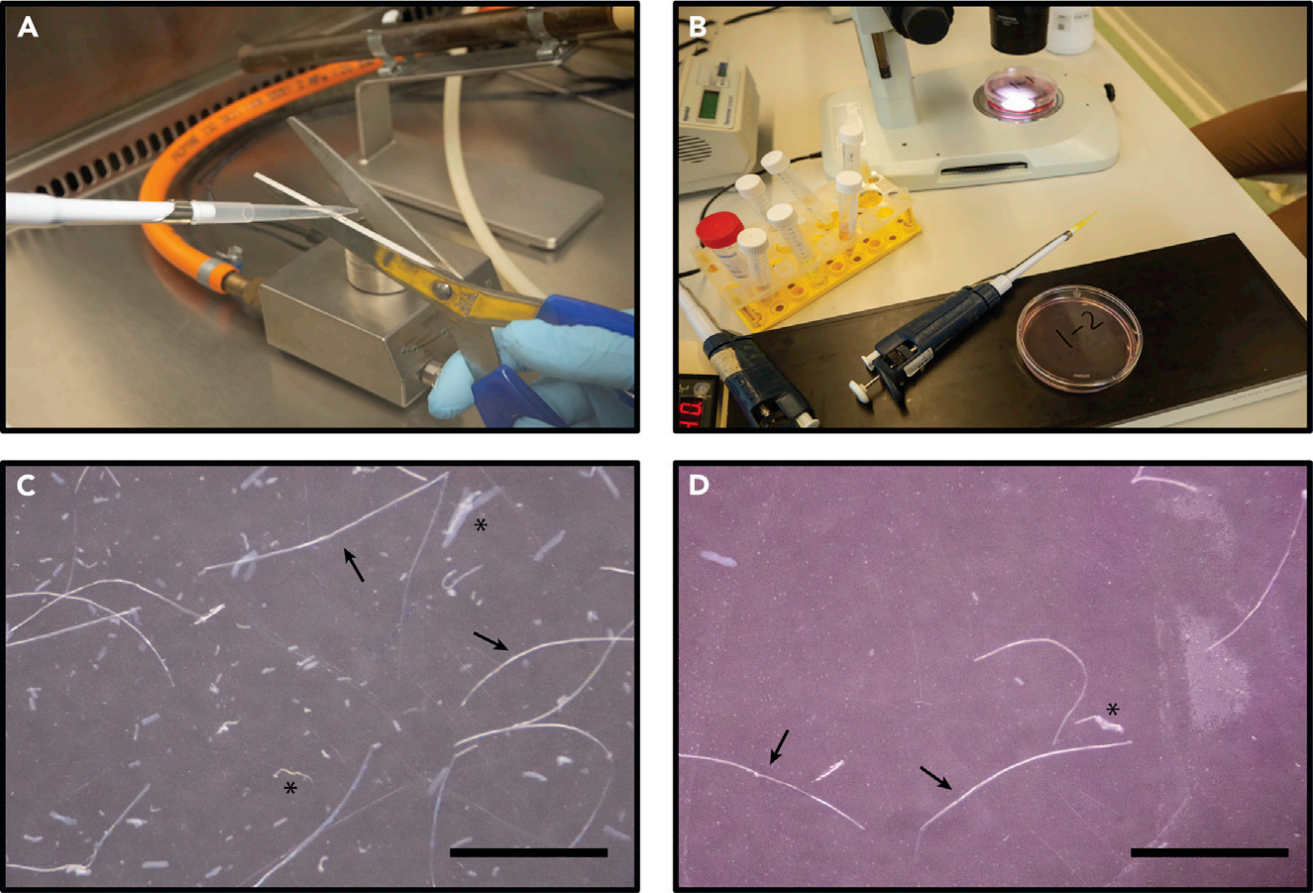
Setup of the material for purification (A) Cutting and polishing pipet tips; (B) Fiber purification setup with slide warmer (bottom), dissecting microscope and two sequential dishes containing fiber medium; (C) fibers in early dish (dish #1-1) with fiber fragments and debris. Arrows indicate viable fibers. Asterisks indicate non-viable fibers/fiber fragments; (D) purified fibers in late dish (dish #1-3). Arrows indicate viable fibers. Asterisks indicate non-viable fibers/fiber fragments. Scale bars, 2 mm.
  d. Pipet up/down with HS before using a new tip to coat it and prevent adhesion of muscle fibers to the inside of the tip.
  e. Release myofibers by pipetting EDL up/down using a P1000 pipette.
    **CRITICAL:** Aspirate and eject the EDL along the length axis; pipet in a smooth motion (Methods video S1). Too much force will damage the myofibers and decrease the yield and viability of the isolated fibers. After the EDL breaks down in smaller parts, move the EDL parts along the length axis through the pipette tip (Methods video S2).
    Methods video S1: Liberating myofibers from digested EDL muscle, related to step-by-step method details step 10 The video shows the first aspirating and releasing steps with the digested EDL. This step is performed with the dish on the slide warmer.
    Methods video S2: Liberating individual myofibers from digested EDL fragments, related to step-by-step method details step 10 The EDL has been broken down into smaller parts and fibers are released from the individual parts. This step is performed with the dish on the slide warmer.
  f. Continue for maximally 10 min., then place dish #1-1 back in the 37°C incubator. Leave the dish for at least 15 min to recover (See troubleshooting – problem 2).
  g. Perform step-by-step method details steps 10a-f for EDL2 in a new dish (dish 2-1).
  h. While myofibers released from EDL2 are recovering repeat step-by-step method details steps 10f-g for dish #1-1.

10. Liberate fibers from the digested muscles.
  a. Take purification dish #1 (dish 1-1) for the first EDL (EDL1) from incubator and place on slide warmer.
  b. Empty tube with EDL in dish #1-1.
  c. Cut ±2 mm from the top of a sterile 1 mL plastic pipet tip (Fisher Scientific cat # 22170403) and polish by carefully passing the pipet tip through the flame (Figure 2A) (See troubleshooting – problems 1 and 2).
    ***Optional:*** It may not be possible to fit the dissection microscope and the slide warmer inside the laminar flow cabinet. To work outside the laminar flow cabinet, prepare a clean curtailed/polished 1 mL pipet tip by pipetting up/down, respectively, EtOH, PBS and HS. The pipette is now ready to use. Repeat this step each time you insert the pipette tip in medium again.

Alternate between dish #1-1 and #2-1 until no more fibers release.

***Note:*** Although isolated viable myofibers can reach up to 5 mm in length, they are typically 1.5–3 mm long, transparent, with smooth sarcolemmal surface when visualized using phase contrast microscopy (Figure 2). However, it is unclear whether isolated fibers are intact fibers or whether they self-seal (Järvinen *et al.*, 2008). Cross-striations and protuberant peripheral nuclei are visible at 200× magnification. Contracting, dying, and damaged fibers are short, opaque, and often curved.

11. Sequentially select viable myofibers.
  a. Place dish #1-1 (with fibers) under the dissection microscope and dish #1-2 (empty dish with warm fiber selection medium) on the slide warmer (Figure 2B).
  b. Curtail and flame-polish a 200 μL tip (Fisher Scientific cat # 10739254).
    ***Note:*** The sharp edges left after cutting the tips using scissors may damage the fibers while pipetting up and down. Polishing using a flame will smoothen the edges of the tips for successful isolation of viable myofibers.
    ***Optional:*** When working outside a laminar flow cabinet clean pipet tip through EtOH, PBS, and HS as described above. Repeat this for each new pipette tip.
  c. Transfer transparent, elongated, straight myofibers from dish #1-1 to dish #1-2 using a P200 pipette (Methods video S3 and S4). Avoid contracted and opaque fibers, fragments, and adipose tissue (Figure 2C).
    Methods video S3: Transferring myofibers during purification, related to step-by-step method details step 11 Transferring myofibers from the source dish (e.g., dish 1-1) to the next purification dish (e.g., dish 1-2). The source plate is under the dissecting microscope at the top of the video, the next purification dish is on the slide warmer at the bottom of the video.
    Methods video S4: Transferring a single myofiber along the length axis, related to step-by-step method details step 11
  d. Continue selecting myofibers for maximally 10 min before returning the dishes to 37°C incubator. Leave the dishes for (at least) 15 min to recover.
  e. In the meanwhile, perform step-by-step method details steps 11a-d for dish #2-1 and #2-2.
  f. When dish #1-1 contains no more intact myofibers, repeat step-by-step method details steps 11a-d to transfer myofibers from dish #1-2 (under dissecting microscope) to dish #1-3 (on slide warmer). The same applies for EDL2: when dish #2-1 is empty, start transferring myofibers from dish #2-2 to #2-3.
  g. When dishes #1-3 and #2-3 contain no more viable myofibers (Figure 2D), move fibers to the dish with experiment base medium (dishes #1-4 and #2-4, respectively).
    ***Note:*** Experiment base medium is medium used for the actual experiment but without experiment additives such as growth factors, inhibitors, activators etc. These additives have been added to the respective wells during plate preparation (see before you begin step 12).
  h. Continue transferring fibers to dish #1-4 and #2-4 until no more viable myofibers are detected in dish #1-3 and #2-3, respectively.

***Note:*** When the yield is high (>250 myofibers from 2 EDL muscles), dishes #1-4 and #2-4 may still contain debris and fiber fragments. If necessary, add an extra purification step by introducing a fifth dish (dishes #1-5 and #2-5) with experiment base medium and continue purifying intact viable fibers. A highly pure collection of myofibers is necessary to prevent transferring contaminants to experimental wells.

***Optional:*** If proliferation of non-myogenic cells is observed using this culturing strategy, an alternative plating method can be used to ensure formation of pure myogenic fiber-derived cultures, as described in the Optional Step below.

***Optional:*** Single fibers can be first cultured for 48 h in experiment base medium in dishes coated with 20% HS. This promotes contaminating non-myogenic cells to release from the fibers and to adhere to the dish, while at the same time preventing fibers to adhere to the bottom of the well. After this step, single fibers are replated in the experiment dishes and the protocol can continue as described below (step-by-step method details step 12a)*.*

**Figure 3:**
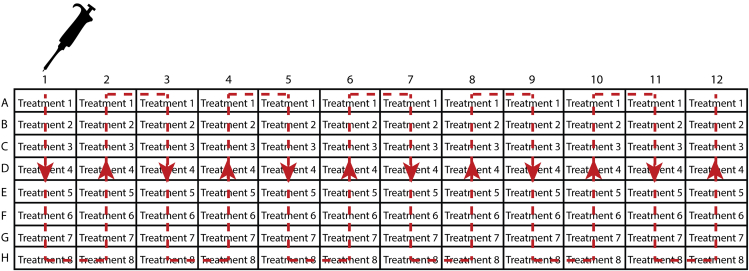
Suggested fiber pipetting scheme Treatments are arranged in rows. Start adding the first fiber to well A1 (treatment 1), the second to well B1, etc. The dashed red line indicates the order of adding fibers to the respective wells.
  f. Continue with a second set of dishes or wait 15 min before resuming picking intact myofibers.
  g. Incubate the myofibers in the respective treatments for desired times. Typically, myofiber-derived colonies can be observed within 72 h after start incubation. Treatment time depends on background strain and selected treatments (See troubleshooting – problem 3).

12. Plating myofibers for assessment of satellite cell activation.
  a. Place the 96-well plate prepared at before you begin step 12 in an incubator at 37°C for at least 15 min to warm.
  b. Place the 96-well plate on slide warmer.
  c. Use a freshly cut and polished tip (cleaned with EtOH, PBS and HS as described at the note before step-by-step method details step 10) to select a single viable myofiber in 50 μL experiment base medium from dish #1-4 (EDL1) or #2-4 (EDL2), and transfer to a 96-well containing 50 μL experiment medium (i.e., medium with 2× concentrated treatment).
    ***Note:*** As mentioned in before you begin step 12b, the respective treatments in the 96-well plates are 2× concentrated and will be diluted to the appropriate concentration automatically by adding the myofiber in medium.
    **CRITICAL:** Avoid transferring any debris with the myofibers, as this may affect the experiment outcome (Tsuchiya *et al.*, 2020).
  d. Pick the next fiber and allocate to a well containing the next treatment (i.e., next row in the plate layout).
    ***Note:*** We recommend adding a single myofiber to the first well of each treatment before transferring a myofiber to the second well per treatment (first well of next column). This approach allows equal and random distribution of myofibers across all treatments and minimize selection bias (Figure 3).
  e. After 10 min return the 96-well plate and fiber-containing dish to the incubator.

### Fixation and immunostaining myofibers

Timing: 5 h

13. Fixing treated myofibers.
  a. Pre-warm 8% PFA (v/v) at 37°C.
  b. Place 96-well experiment dish with cultured fibers on slide warmer.
  c. Add 100 μl of prewarmed 8% PFA (v/v) to each well and incubate for 15 min.
    **CRITICAL:** This step has to be performed inside a chemical safety cabinet to contain toxic fumes.
    ***Note:*** After incubating 15 min in PFA, plates can be further processed at 18°C–22°C .
  d. Aspirate PFA solution and replace with PBS.

***Note:*** Fixed fibers can be stored at 4°C up to 2 weeks or stained directly.

**CRITICAL:** Rinse wells thoroughly with PBS to remove all PFA. This is to prevent over-fixation, as this will compromise subsequent immunostaining and may require antigen retrieval approaches.

**Figure 4:**
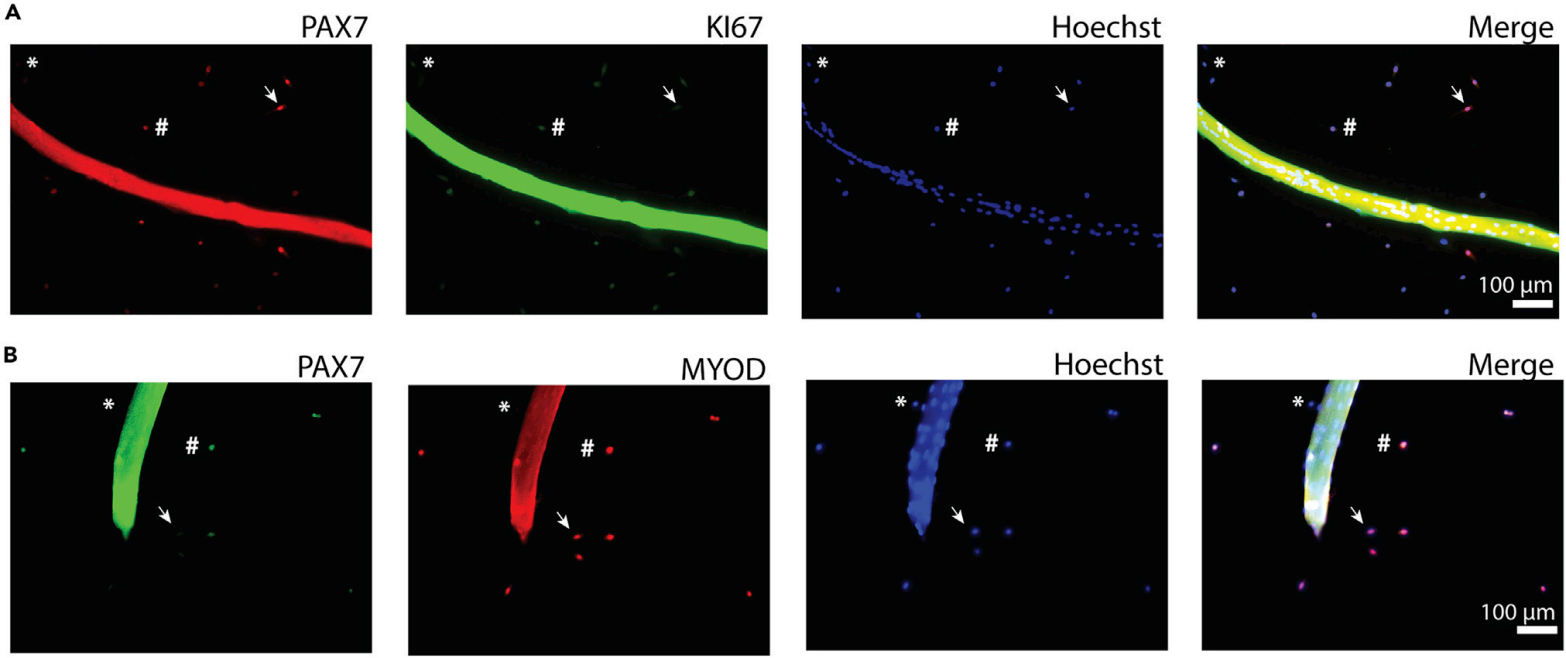
Immunostaining of PAX7/KI67 and PAX7/MYOD in myofibers (A) Myofibers were isolated from GAAKO donor animals and cultured for 72h in proliferation medium. ∗ indicates a PAX7-low/KI67^+^ cell; # indicates a PAX7^+^/KI67^+^ cell; arrow indicates a PAX7^+^/KI67-low cell. (B) Myofibers were isolated from WT FVB/N donor animals and cultured for 72h in proliferation medium. ∗ indicates a PAX7^-^/MYOD^-^ cell; # indicates a PAX7^+^/MYOD^+^; arrow indicates a PAX7^-^/MYOD^+^ cell. Red indicates MYOD; green indicates PAX7; nuclei were counterstained with Hoechst.
  b. Block 30 min with 20% HS (v/v).
  c. Incubate with anti-PAX7 primary antibody (1 in 100 in PBA-Tw) for 1 h at 18°C–22°C .
    ***Optional:*** Previous step may be performed 12–16 h at 4°C in a humidified chamber.
  d. Rinse once with PBS-Tw.
  e. Incubate with anti-KI67 primary antibody (1 in 100 in PBA-Tw) + biotinylated anti-mouse IgG (1 in 250 in PBA-Tw) for 1 h at 18°C–22°C .
    ***Note:*** For PAX7-MYOD co-staining, incubate with anti-MYOD primary antibody (1 in 500 in PBA-Tw) + Biotin Anti-Mouse IgG1 (1 in 250 in PBA-Tw) for 1 h at 18°C–22°C .
  f. Rinse once with PBA-Tw.
  g. Incubate with Streptavidin-AF647 (1 in 500 in PBA-Tw) + goat anti-rabbit AF488 (1 in 500 in PBA-Tw) 1 h at 18°C–22°C .
    ***Note:*** For PAX7-MYOD co-staining, incubate with Streptavidin-AF647 (1 in 500 in PBA-Tw) + goat anti-mouse IgG2B-Cy3 (1 in 500 in PBA-Tw) 1 h at 18°C–22°C .
  h. Rinse once with PBA-Tw.
  i. Incubate with Hoechst (at 1 μg/mL in PBS) for 15 min at 4°C.
  j. Rinse once with PBS-Tw.
  k. Add 100 μl PBS/well to keep cells moist.
  l. Image immunostained cells as soon as possible for the best results, but at least within one week after finishing immunostaining.

14. Immunostaining fiber-derived cells.
  ***Note:*** Activation of myogenic cells can be measured assess in several manners**.** Here we describe a PAX7 and KI67 co-staining that allows to assess proliferative, thus activated, myofiber-derived cells. (Figure 4A). Additionally, we describe a PAX7 and MYOD co-staining that allows a complementary method to assess activation of myogenic cells (Figure 4B). Other combinations of antibodies are possible (See troubleshooting – problems 4 and 5).
  a. Permeabilize with 0.5% Triton in PBA (see Table materials and equipment for abbreviations; v/v) for 30 min at 18°C–22°C.

## Expected outcomes

### Myofiber yield

The yield of viable myofibers is dependent on the age, background strain, and genetic makeup of the donor mice. The sex of the donor may affect myofiber yield as well, but we did not test this. Young adult FVB/N animals (8–12 weeks of age) will yield ±250 intact myofibers and the yield is reduced to <150 myofibers in FVB/N mice of ≥30 weeks. Myofibers from wild type FVB/N mice can be kept for a week in culture under the conditions described in this protocol. Maximum culture times should be tested for other genetic backgrounds and disease models.

Isolating myofibers from animals with a muscle-degenerative condition will affect the number and quality. We have wide experience with animals that are knockout for acid alpha glucosidase (GAA), i.e., the mouse model for Pompe disease that was generated in our laboratory (Bijvoet et al., 1998). GAAKO mice, which are on a FVB/N background, develop a muscle phenotype after 15 weeks of age (Schaaf *et al.*, 2018). Myofiber yield from young FVB/N and GAAKO animals is similar, but myofiber yield from GAAKO donor mice of 15 weeks and older is reduced compared to age-matched wildtype FVB/N donor mice. GAA-deficient myofibers have a fragile morphology and show accumulation of debris in the core of the fibers as result of distorted autophagy in GAA-deficient myofibers (Lim *et al.*, 2015).

### Size of colonies of myofiber-derived cells

**Figure 5:**
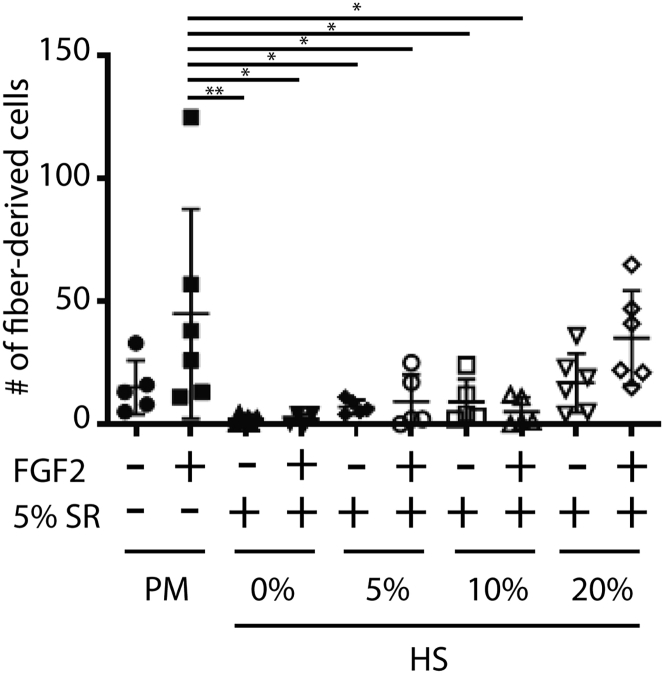
Identifying conditions that activate satellite cells from specific donor backgrounds Myofibers were isolated from WT FVB/N donor animals and cultured for 72h under indicated conditions. PM: proliferation medium (Ham’s F10/20 % FCS); SR: knockout serum replacement. Data are indicated as mean ± SE. Statistics by one-way ANOVA followed by Tukey correction for multiple testing. ∗p<0.05; ∗∗p<0.01. n=4-6.

The formation of colonies from myofiber-derived cells is dependent on host factors, such as age and genetic makeup of the donor, as well as on culture conditions that are used. The satellite cell response assessed with this assay is a relative measure, i.e., is compared to the colony size under basal conditions. Basal conditions are defined experimentally to use media formulations (i.e., experiment base medium) that limit colony formation (i.e., that keeps satellite cells quiescent), but that allow expansion of myofiber-derived cells after adding supplements that stimulate proliferation, such as high concentrations of serum, FGF2 or HGF. Figure 5 shows the results from such an experiment defining the experiment base medium for myofibers isolated from FVB/N donors. Using defined conditions, the relative potential of satellite cells from different disease models or treatments can be determined.

### Myogenic profile of myofiber-derived cells

All myofiber-derived cells are progeny of satellite cells and should express PAX7 and/or the myogenic regulatory factors MYOD or MYOG in the first week of culture. Immunostaining of the colonies is then used to verify the myogenic identify of the cells and is valuable as a quality control of the purity of myofiber selection and plating. In order to ensure best results wells containing >10% non-myogenic cells should be excluded from analysis. Nevertheless, we can not rule out that this will be different in disease models. In our experience four rounds of purification are sufficient to ensure >90% pure myogenic cultures (Figure 4B). However, if necessary, additional purification steps can be added. In addition, as suggested as an optional step in step-by-step method details 12, pre-culturing fibers in non-adhering conditions may increase the purity of myogenic colonies during the experiment.

## Quantification and statistical analysis

**Quantifying myofiber-derived cells**: After fixation, the immunostained myofibers/cells are imaged. Exclude wells that contain short myofiber fragments, contracted or damaged fibers as these may affect the outcome through release of satellite cell activation signals (Tsuchiya *et al.*, 2020). The expression of myogenic markers is used to quantify colonies containing satellite cell progeny. The Hoechst (blue) signal is used to identify and count all nuclei. Our analyses indicate a purity >90% of myogenic colonies when the purification steps are followed as described in this text. It is advised to check contamination with non-myogenic cell types in the initial experiments, for instance, by immunostaining. Different markers can be used and are commonly described in the literature to detect fibroadipogenic cells (FAPS), endothelial cells, and pericytes. However, in order to reduce the possibility of including non-myogenic cells in the analysis, we typically include only those cells that are growing adjacent to the myofiber and exclude cells present at a distance farther of 200 μm from the fiber. As a rule of thumb, this is facilitated by including part of the myofiber in each image when using a 10× objective. Image the direct perimeter around the myofiber by taking sequential images. In this protocol a Nikon Eclipse Ti with a 10× objective was used, but equivalent setups may be used. Typically, 5–6 images per myofiber are taken to cover the whole length of the myofibers. For each condition myofiber-derived cells from at least 5 different intact myofibers are counted manually. Colony size is expressed as the number of myofiber-derived cells per myofiber.

***Alternatives:*** Image analysis software such as FIJI or Adobe Photoshop can be used to automate quantification.

## Limitations

The protocol described here is suitable to identify fiber-derived myogenic cells and to assess their state of activation. However, this method is not suitable to determine the origin of the colonies. For such purpose, a lineage tracing strategy would be more adequate.

Other strains or mice from other ages: we have used this protocol mainly for isolating myofibers from adult mice aged between 8 and 40 weeks. We obtained reproducible yields of myofibers also from 40 weeks GAAKO donor animals, which have already developed a considerable muscle phenotype at that age. Using the protocol for other strains, including transgenic lines, older/younger mice should be verified and may require optimization of digestions parameters (collagenase type II concentration, digestion time, plating media).

Other muscles: we did apply this protocol successfully to isolate myofibers from soleus and diaphragm muscle (not shown), but cannot exclude that using the protocol to obtain myofibers from other muscles require optimization. Most likely, it requires developing a dissection approach for the muscle of interest. The dissection strategy in this protocol is designed for muscles with easy identifiable tendons, such as the EDL. The EDL is then dissected at/through its tendons without damaging the myofibers. Damaging myofibers reduces the yield and viability of the isolated fibers.

## Troubleshooting

### Problem 1

Few viable (transparent) myofibers obtained (step-by-step method details step 11).

### Potential solution

Solution 1: Improve dissection technique: swift but careful dissection of the EDL muscles is key to the success of myofiber isolation and takes practice to master and produce consistent yields. Prevent stretching and damaging the muscles. Ensure cutting the tendons to release the EDL and not to damage the myogenic part of the muscle. Limit cooling the muscles by prewarming the digestion solution.

Solution 2: Check digestion solution. The quality of collagenase type II batches may vary. Ensure to make sufficiently large stock solutions to finish the experiments dedicated to specific projects. Stock solutions can be stored at −80°C up to 6 months in our hands with negligible loss of yield. We advise to try collagenase everytime a new stock is made in order to adjust calculations if myofiber yield was lower than expected.

Solution 3: Check digestion time: over/under digestion will result in variable myofiber yield and quality. The optimal time may differ slightly per collagenase type II stock solution.

Solution 4: Handle myofibers more carefully: make sure all pipet tips are polished properly, avoid forcing the fibers through the tips or bending fibers during handling.

Solution 5: If possible, preferentially select longer myofibers (>1.5 mm), as short myofibers are usually damaged and will not survive.

### Problem 2

Many contracted myofibers (step-by-step method details step 11).

### Potential solution

Solution 1: Practice to improve dissection technique. Success of myofiber yield (number and quality fibers) is largely determined during dissection.

Solution 2: Prevent cooling the myofibers by returning the dishes to the incubator within 10 min and allow to recover for at least 15 min between myofiber selection sessions.

Solution 3: Handle myofibers more carefully: make sure all pipet tips are polished properly, avoid forcing the fibers through the tips or bending fibers during handling.

### Problem 3

Few myofiber-derived cells (step-by-step method details step 12).

### Potential solution

Solution 1: Optimize culture conditions, predominantly the media composition as described in materials and equipment. Define media conditions to obtain a maximal increase in the number of myofiber-derived cells after adding 20 ng/mL FGF2 for at least 72 h.

Solution 2: Verify that viable myofibers have been obtained. One could consider to add viability dyes such as trypan blue, but verify that these do not interfere with the experiment’s objective. A retrospective method entails staining myofibers after PFA fixation for Hoechst. Lack of nuclei staining indicates loss of myofiber viability somewhere along the process. Optimize myofiber isolation technique before planning a new experiment.

Solution 3: Extend culture time as satellite cells from some donor strains or genetic backgrounds, including wild type FVB/N, display slow/delayed activation response.

### Problem 4

Lack of staining for selected markers (step-by-step method details step 14).

### Potential solution

Solution 1: Verify critical steps of staining protocol: proper permeabilization (over/under permeabilization negatively affect staining of nuclear proteins); (primary and secondary) antibody concentration. Optimize staining protocol on primary satellite cell-derived cultures.

Solution 2: Ensure using high-resolution optics to allow imaging low signals. This includes use of imaging-compatible culture plates (e.g., Nunc Nunclon 96-well plate) (see key resources table).

### Problem 5

Weak PAX7 signal in immunostaining (step-by-step method details step 14).

### Potential solution

Solution 1: Fix fibers as described for 15 min and not longer than this. Overfixation could lead to increased background in the immunostaining or even mask the antigens.

Solution 2: Incubate with primary antibody anti-PAX7 8–12 h at 4°C. in a humidified chamber. Proceed with the rest of the immunostaining as described.

Solution 3: Culture fibers during different time points (e.g., 24, 48, 72, 96, and 120 h), fix, and stain in order to calculate the time window during which PAX7 is expressed and to prevent loss of PAX7 expression due to satellite cells transitioning into a myoblast state (PAX7^-^/MYOD^+^).

Solution 4: Culture fibers under different quiescence/activation conditions (e.g., varying the concentration of serum) to determine the most optimal for your desired experiment.

## Resource availability

### Lead contact

Further information and requests for resources and reagents should be directed to the lead contact Dr. Gerben Schaaf at g.schaaf@erasmusmc.nl

### Materials availability

This study did not generate new unique reagents.

### Data and code availability

This study did not generate/analyze any datasets/code.
